# Notes and News

**Published:** 1890-07-26

**Authors:** 


### Notes and News.

Collected and Communicated.

St. Raphael's, Worthing, which was specially set aside for consumptives, April 1st, 1889, has admitted 162 patients for treatment during the year, and in consequence of the daily increasing number of applications for admission, it has been decided to extend the hospital. To this end adjacent premises have been secured and will shortly be ready for the reception of consumptive women and girls. The Charity Organisation Society of London retain six summer and three annual beds.

The committee of the Zenana Medical College, 58, St. George's road, have much pleasure in announcing to their friends and supporters that H.R.H. the Duke of Connaught, late commander of the troops in the Bombay Presidency, and who wrote, "I am sure that these means of educating native ladies to understand the rudiments of medicine so as to be able to treat the numerous cases that occur in zenanas will do more to elevate them than anything else," has graciously consented to become president of the College.

The programme of the tenth International Medical Congress, to be held at Berlin, has been published. Some hundreds of prominent foreign physicians will attend. Sir Joseph Lister will speak on the present position of antiseptic surgery. Dr. Koch, who first drew attention to the cholera bacillus, will follow with a lecture on bacteriology. Professor Wood, of Philadelphia, will lecture on anæsthetics, and many other distinguished members of the profession are also expected to deliver lectures.

At a meeting of the directors of Kilmarnock Infirmary, the secretary, Mr. D. C. Gairdner, intimated having received a letter from the Dowager Lady Scott Howard de Walden, Lady of the Manor, conveying her desire to present the institution with the sum of £1,200 to enable the directors to erect a children's ward, and also to provide increased dormitory accommodation for the nurses and domestics. On the motion of the Chairman, ex-Bailie Craig, it was unanimously agreed to tender her ladyship a hearty vote of thanks for this very generous and munificent gift, and the secretary was desired to ascertain if her ladyship had any objections to the new ward being entitled "The Scott Howard de Walden Children's Ward."

The forty-ninth annual report of Cumberland Infirmary states that 723 in-patients and 1,576 out-patients were treated last year. The net expenses were £3,671. All the tables are very full and clear, and reflect credit on the secretary; the expenses seem well kept down, especially in the housekeeping department. The extraordinary expenditure was rather high last year, amounting to £1,489.

Many of our readers at Addenbrooke's and the London have fresh before their minds the sad death of Mr. R. Gerard Wilde. A cot has been endowed in his memory in the children's ward of the London Hospital, and now his sister is about to erect a mortuary chapel at Addenbrooke's, also to his memory. Miss Wilde is a staff-nurse at Addenbrooke's, and her action receives the sympathy and support of the committee. The simple unselfishness of Mr. Wilde's life, and the complete absence of self-consciousness which characterized his many acts of kindness, make it eminently fitting that such a sweet memory should be perpetuated in those two institutions where his short work was fulfilled.

The annual visitation of the Royal Sea Bathing Infirmary, Margate, will take place at Margate, on Monday next, the 28th inst. There will be a short service in the chapel, at two p.m., and the public meeting afterwards, the wards being open for inspection during the afternoon. It is very desirable that there should be a large attendance of the governors of this most necessary charity, so that they may satisfy themselves that the sanitary and other arrangements are what they ought to be in all respects.

The advantages of meat essences in travelling are proved in many places "In Darkest Africa." Take the following instance as a specimen: "On the 22nd, soon after the advance had reached camp, a cold and heavy shower of rain fell, which demoralised many in the column; their failing energies and their impoverished systems were not proof against cold. Madis and Zanzibaris dropped their loads in the road, and rushed helter-skelter for the camp. One Madi managed to crawl near my tent, wherein a candle was lit, for in a rainstorm the forest, even in daylight, is as dark as on an ordinary night in the grassland. Hearing him groan, I issued out with the candle, and found the naked body rigid in the mud, unable to move. As he saw the candle flame his eyes dilated widely, and he attempted to grasp it with his hands. He was at once borne to a fire, and laid within a few inches of it, and with the addition of a pint of hot broth made from the Liebig Company's Extract of Meat we restored him to his senses. On the road in front of the rear guard two Madis died, and also one Zanzibari of the rear-column, stricken instantaneously to death by the intensely cold rain."

And while writing about this much-read book we must quote the tribute paid in the first chapter to our great chemists: "Messrs. Burroughs, Wellcome, and Co., of Snow Hill Buildings, London, E.C., the well-known chemists, furnished gratis nine beautiful chests replete with every medicament necessary to combat endemic disease peculiar to Africa. Every drug was in 'tabloids' mixed with quick solvents; every compartment was well stocked with essentials for the doctor and surgeon. Nothing was omitted, and we all owe a deep debt of gratitude to these gentlemen, not only for the intrinsic value of these chests and excellent medicines, but also for the personal selection of the best that London could furnish, and the supervision of the packing, by which means we were enabled to transport them to Yambuya without damage."

The following vacancies are announced this week, the amount of the salary in each case being given after the name of the institution :—

**Resident Medical Officers.**

Birmingham General Hospital, two Assistant House Surgeons—board, etc.

North Staffordshire Infirmary, Assistant House Surgeon—board, etc.

Metropolitan Asylums Board, Clinical Assistant for Darenth Schools—board, etc.

Counties of Caithness and Sutherland, Medical Officer of Health —£500.

The Council of the Metropolitan Hospital Sunday Fund meets on Monday next at the Mansion House to order the payment of awards to hospitals and dispensaries as recommended by the Committee of Distribution.

In Lord Brassey's Indian Museum, the first annual meeting of the National Association for Supplying Female Medical Aid to the Women of India was fitly held. Colonel Robertson, the honorary secretary, read a report, in which it was stated that the operations of the association had now extended throughout the entire Indian Continent. Thirty lady doctors and assistant surgeons (of whom eight lady doctors had been sent from England) were at present working in connection with the Countess of Dufferin's fund, and 238 female pupils were studying at Indian medical schools. The position of women in India formed the subject of discussion at an influential meeting held on the same day at the residence of Mr. and Mrs. Francis Jeune.

The report of the Special Committee for remedying the defects of Derby Infirmary will be submitted to the governors on the 29th. The following are the recommendations: (1) That the most satisfactory course would be to erect an entirely new hospital, on the present site, but to the north or south of the present building; (2) that an alternative, but less satisfactory, course would be to retain and remodel the Nightingale wing, the kitchens, and operating theatre, and erect the required new building adjoining thereto; (3) that your committee be reappointed, with power to add to their number, to draw up conditions and requirements of new buildings; (4) that plans be obtained from not less than three architects of experience in hospital construction, who can refer to works recently executed by them.

Sir Henry Roscoe, M.P., lately delivered prizes and certificates of honour to the successful students of the medical school of St. Mary's Hospital, Paddington. In addressing a large audience, he said that nothing would do more to raise the status of English medicine than the formation of research scholarships of moderate amount, to be given to the various medical schools in London, and it was one of the first duties of the great medical corporate bodies to assist in widening the boundaries of their sciences. Were the medical authorities satisfied with the present state of things? He feared that this might to some extent be the case, and that the introduction of what might be termed new-fangled ideas did not meet with much sympathy. Yet the world moved on, and those who did not go forward would be left behind. Neither the chemist nor the physiologist was able to explain the anæsthetic effects of chloroform any more than the remarkable but well-attested phenomena of hypnotism. The scholarships announced consisted of entrance scholarships in natural science of fifty guineas each to Mr. A. E. Wilson, Mr. H. J. K. Banfield, Mr. A. Stanley, and Mr. A. W. Sanders; University entrance scholarships of the same amount to Mr. E. A. Shaw, B.A., and Mr. A. Senior, B.A.; an Epsom scholarship of the value of £105 to Mr. H. J. Lawrence, a classical scholarship of £50 to Mr. E. G. Mayo, and an exhibition in natural science of £25 to Mr. E. Morgan.

Sir Richard Wallace, K.C.B., who died somewhat suddenly last Sunday, was a noted philanthropist in Parisian circles. After the war of 1870, in addition to organizing a fund for the relief of the destitute British subjects remaining in the capital, he devoted £12,000 to the founding of an ambulance, known as the Hertford Ambulance, which was administered by the Société de Secours aux Blessés; he also opened a special ambulance in his own house. After the siege he took a leading part in the work of revictualling the starving French capital; and has left a lasting monument in the many hundred drinking-fountains to be seen all over Paris. Many years ago he erected at Neuilly the Hertford Hospital, for the special benefit of British subjects, a model establishment, not only from its arrangements, but also from the admirable system under which it has been managed. At the time of his death Sir Richard was making arrangements to transfer the Hertford Hospital to the British Government with an endowment of £150,000. He also gave £100 to the National Pension Fund for Nurses.

The total number of reported lunatics on the 1st of January last is made up as follows: 8,095 (4,040 male and 4,055 female) private patients, excluding criminals; 77,257 (34,374 male and 42,883 female) pauper patients; and 715 (545 male and 170 female) criminal patients. These figures show, as compared with the 1st January, 1889, a decrease of 12 male and an increase of 137 female private patients; an increase of 538 male and 1,087 female pauper patients, and a decrease of 22 male and one female of the criminal class. The total increase of the year is 30 higher than it was in 1889, but is 25 lower than in 1888. The ratio per 10,000 of reported lunatics, idiots, and persons of unsound mind to the population on the 1st January of the years is given from 1859 to 1890. The increase during the past year has been from 29 07 to 29·26, or ·19 per 10,000 of the population. This increase has been entirely among the pauper class, and is said to be due, not to increase of insanity, but to the accumulation of the insane in the pauper asylums.

On the 10th inst. Dr. Simon, assistant physician to the General Hospital, was examined at Birmingham by the Hospital Reform Committee. What with committees at Birmingham, Westminster, and Melbourne, the subject of hospital abuse is being thoroughly thrashed out at last. Dr. Simon said he had occupied his position at the hospital for 11 years. His duties were confined to the attention of out-patients. He thought he never saw less than 100 patients a day, but as a rule the number was from 250 to 300. He was occupied at the hospital on the average from three to four hours a day. During his experience there had been a great increase in the number of out-patients; in fact, there had been an increase in all directions. He thought the abuse of the hospitals by those who could afford to pay physicians' consulting fees was very small. If visited at the patient's home the physician's fee was generally two guineas, but at the physician's chambers the usual charge was one guinea. He thought that quite 50 per cent. of the cases that went to the hospitals were of a trivial character. He suggested that persons should consult their own doctor or attach themselves to some institution by a fixed payment. Of course he could not suggest a practical remedy for the evil complained of, but was of opinion that the public should provide for medical attendance at their own cost. Dr. Simon seemed to think the cases ought to be sifted out by a junior before they came to him; the waste of his time was enormous. In answer to the Chairman, Dr. Simon said he did not think it quite fair to the profession under existing circumstances that patients should pay for admission to public hospitals when the fee paid for admission went to the hospitals.

